# Add-on unidirectional elastic metamaterial plate cloak

**DOI:** 10.1038/srep20731

**Published:** 2016-02-10

**Authors:** Min Kyung Lee, Yoon Young Kim

**Affiliations:** 1 Department of Mechanical and Aerospace Engineering, Seoul National University, 1 Gwanak-ro, Gwanak-gu, Seoul, 151-742, Korea; 2 Institute of Advanced Machine and Design, Seoul National University, 599 Gwanak-ro, Gwanak-gu, Seoul, 151-744, Korea

## Abstract

Metamaterial cloaks control the propagation of waves to make an object invisible or insensible. To manipulate elastic waves in space, a metamaterial cloak is typically embedded in a base system that includes or surrounds a target object. The embedding is undesirable because it structurally weakens or permanently alters the base system. In this study, we propose a new add-on metamaterial elastic cloak that can be placed over and mechanically coupled with a base structure without embedding. We designed an add-on type annular metamaterial plate cloak through conformal mapping, fabricated it and performed cloaking experiments in a thin-plate with a hole. Experiments were performed in a thin plate by using the lowest symmetric Lamb wave centered at 100 kHz. As a means to check the cloaking performance of the add-on elastic plate cloak, possibly as a temporary stress reliever or a so-called “stress bandage”, the degree of stress concentration mitigation and the recovery from the perturbed wave field due to a hole were investigated.

Although theories for cloaking have been established by Pendry *et al.*^1^ and Leonhardt^2^ for electromagnetics and by Milton^3^ for elasticity, the progress in metamaterial cloaks, especially in elastic cloaking devices, is still in its infancy. (See also, for instance refs. 4, 5, 6, 7 for metamaterial realizations in electromagnetics and acoustics). The objective of this study is to present experimental results for elastic cloaking by proposing an unprecedented concept of an “add-on type” metamaterial cloak. Cloaking an object against elastic waves always requires a device that is in physical contact with a base system containing a target object. Hence, the earlier investigations of elastic metamaterial cloaks^8^,^9^,^10^,^11^,^12^ focused on embedded elastic metamaterial cloaks. This implies that they were realized by machining or fabricating the base structure containing a target object, inevitably causing an undesirable permanent alteration of the original base structure and thereby weakening structural stiffness. Consequently, the embedded elastic metamaterial cloaks are limited in their applicability.

Because metamaterial cloaks are capable of detouring the path of a wave, their potential use as first-aid devices to prevent structural failures due to concentrations of stress caused by the sudden creation of a hole (or a crack) in a thin-walled structure is an important research area. In such a scenario, as embedding a metamaterial cloak in a structure is unrealistic, add-on type elastic cloaks are preferred as stress-relieving devices. Here, an add-on type cloak refers to a new type of cloaking device that can be placed on a base structure surrounding a target object instead of being inserted or embedded in the base structure of a metamaterial cloak. The add-on cloak should be mechanically coupled with the base structure to detour the elastic waves around a target object. To this end, we designed and fabricated an add-on elastic metamaterial plate cloak and performed wave experiments. Thus, we investigated the amount by which an add-on cloak can mitigate the stress concentration around a hole in a plate and also recover the perturbed wave field back to the original un-perturbed wave field by a hole. Secondary stress concentration elsewhere in the plate may be inevitable, if a stress relieving method other than a cloaking device is employed.

When an add-on type cloak device is used, the wave physics of the system composed of the base structure and the add-on metamaterial cloak is different from the wave physics of a metamaterial cloak embedded plate. Hence, we investigated if the wave detouring performance of an add-on metamaterial plate cloak is equal to that of an embedded metamaterial plate cloak. For the investigation, a 3-mm Cu plate with a 1.5 cm radius hole was considered and subjected to a plane-wave incidence of the lowest symmetric Lamb wave (known as the S0 wave mode) centered at 100 kHz. For the experiments, an add-on metamaterial cloak was placed over the hole. The cloak was mechanically coupled with the region of the base material surrounding the hole. Two performance criteria were considered in the course of the experiments: i) the mitigation of the stress concentration around a hole by the application of an add-on type metamaterial cloak and ii) the reduction in the wave field disturbance due to a hole in the cloak.

To design an add-on metamaterial plate cloak, the method of transformation elasticity^2^ using a conformal mapping function was used. The conformal function is frequently used in electromagnetics and acoustics^13^,^14^,^15^,^16^,^17^,^18^,^19^,^20^,^21^,^22^. To date, it has not been used to study in elasticity, perhaps due to the difficulty associated with form-variance^2^. In our metamaterial design, two sets of gradient-refractive-indexed phononic crystals were utilized. Their unit cells were made of a thickness-varying base Cu plate with circular cavities or Si inclusions. Wave measurements in the experiments were conducted by using ultrasonic magnetostrictive transducers^23^,^24^,^25^,^26^. The experiments were performed in the plates for three cases: i) without any cloaking device, ii) with an embedded metamaterial plate cloak and iii) with an add-on type metamaterial plate cloak. With the experimental results, we verified whether an add-on metamaterial cloak functions similarly to an embedded metamaterial cloak as well as if the add-on metamaterial cloak could be used as a first-aid on-site stress reliever that could be immediately applied to a damaged part of the plate structure.

## Results

### Design of Metamaterial Cloak

**Figure 1:**
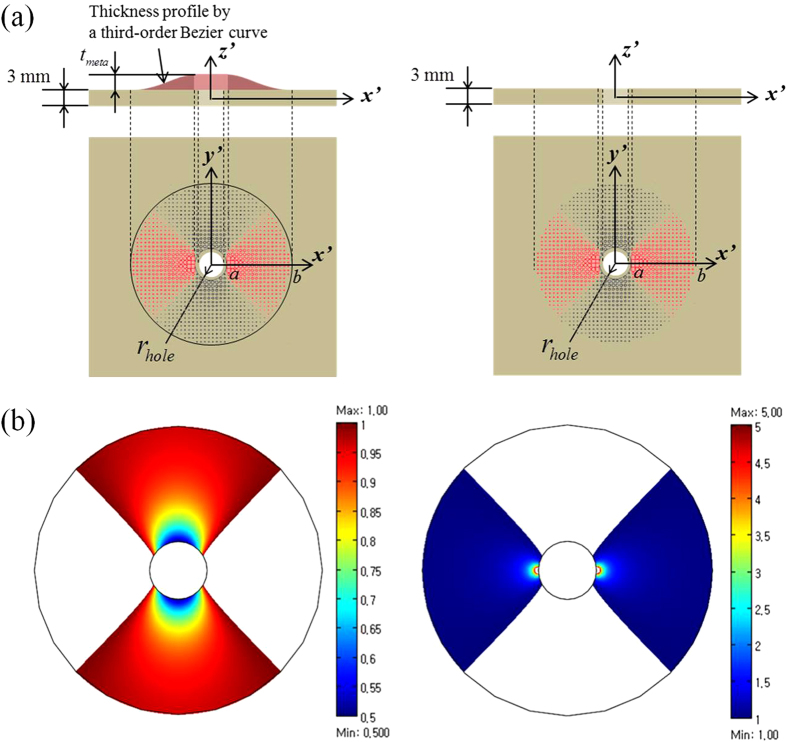
(**a**) The add-on metamaterial cloak installed onto a plate having a circular hole and the embedded cloak in the same plate. (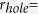
15 mm, 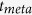 = 3 mm*, a* = 2 mm, *b* = 10 mm, 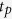 = 3 mm). (**b**) The distribution of 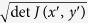 in the annular cloak region. Left: Sub-region 1 (sub-region of 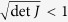), right: Sub-region 2 (sub-region of 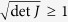).

Figure 1a shows the sketch of an add-on metamaterial cloak installed on a plate with a hole. It is compared with an embedded cloak in a base plate with a hole. Assuming an incidence of a plane symmetric Lamb wave along the 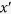
coordinate, we first determined the material properties of an elastic cloak. As the symmetric plane Lamb wave incidence is considered, the two-dimensional plane-stress assumption is used to design the metamaterial cloak, for both add-on and embedded types.

To use the method of transformation elasticity^3^, two Cartesian coordinate systems (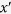, 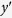, 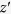) and (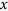, 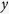, 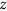) are introduced, where unprimed and primed systems refer to the coordinate systems before and after the transformation. In our notation, the physical space in consideration is described with (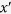, 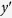, 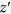).

For an isotropic medium defined in the two-dimensional (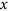, 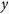) space, the elasticity tensor 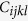 is given by two Lame constants (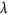, 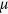) and may be expressed by Eq. (1) as follows:


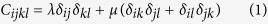


where 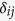 is the Kronecker delta. The chosen conformal mapping function is given by Eqs. (2) and (3) as follows:


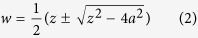


with


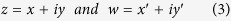


Given Eqs. (2) and (3), it can be shown that the primed metamaterial properties are given by the following:


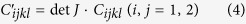



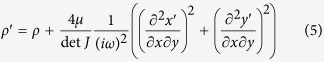



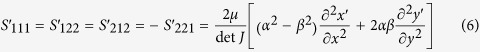



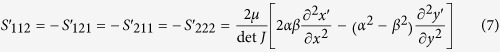



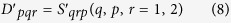


where


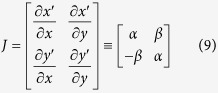



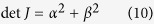


The new terms 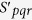 and 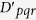
appear in the transformed space (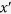, 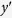) because unlike electromagnetics and acoustics^1^,^2^, the form-invariance property does not hold in the classical elasticity theory^3^. In fact, the governing elastodynamic equations in the transformed space can be expressed as follows:


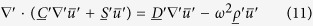


where 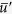 denotes the displacement vector in the transformed space and is related to the displacement vector 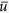 in the original space by 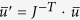, and 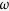 is the angular frequency. The primed gradient implies that the gradient exists with respect to the primed coordinates.

For the selected conformal mapping given in Eq. (2), 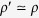 can be assumed at high frequencies, and also, the terms involving 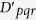
and 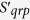 in Eq. (11) may be ignored. Given these assumptions, Eq. (11) may be approximated as follows:


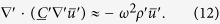


In this case, as illustrated in Fig. 1, the metamaterials can be realized by phonic crystals made of isotropic media. The specific realization will be discussed in the next section.

### Realization of Metamaterial Cloak by GRIN PC

The wave speeds 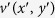 in the transformed space will be compared with 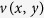 in the original isotropic space, to characterize the metamaterial cloak that is realized by the transformation in Eq. (2) in conjunction with the above-mentioned assumptions. This yielded the following relation:


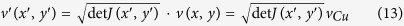


where 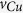 is the lowest symmetric Lamb wave mode speed in a Cu plate at the selected frequency of 100 kHz. Therefore, our task is to fabricate a metamaterial cloak that exhibits the wave speed distribution given in Eq. (13). Figure 1b plots 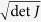, which is the same as 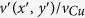. In Fig. 1b, the cloak region is divided into sub-region 1 of 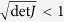, and sub-region 2 of 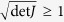. In sub-region 1, the wave velocity was slower than the wave speed (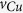) in a Cu plate, and in sub-region 2, the wave speed is faster than 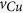.

**Figure 2:**
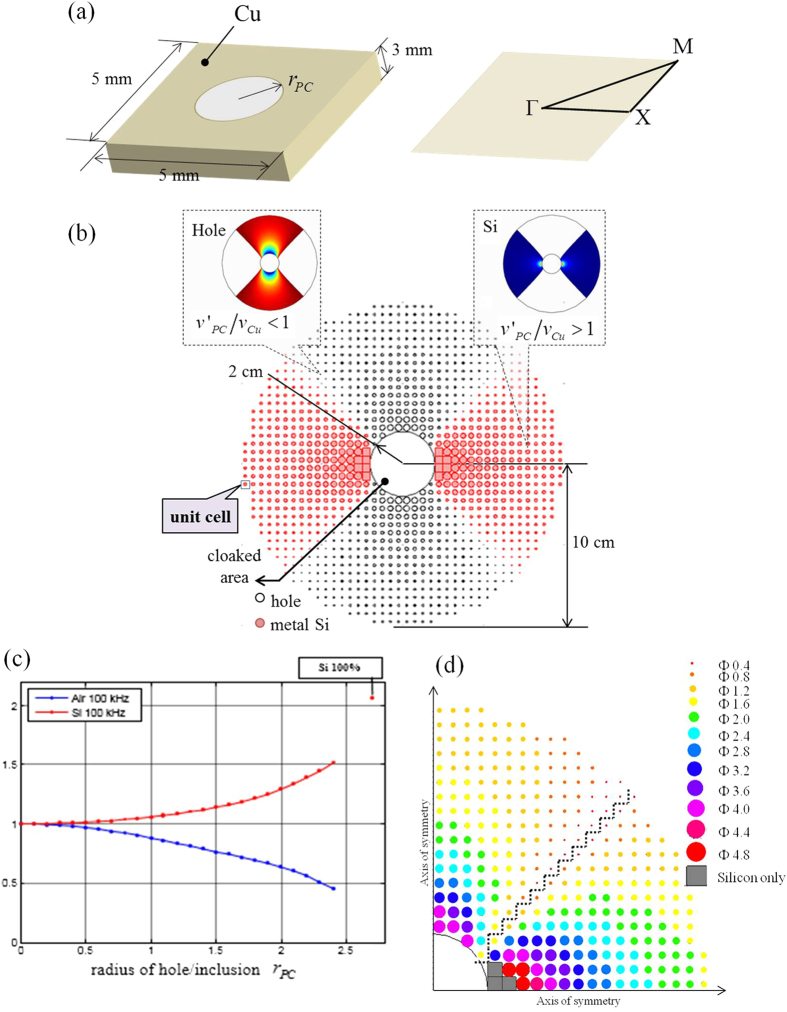
(**a**) The unit cell of the square-lattice phononic crystal with a circular inclusion and the first Brillouin zone of the square lattice illustrated with symbols 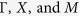, denoting the directions of the wave propagation. The inner and outer radii of the PC cloak are 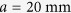 and 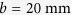, respectively. (**b**) The cloaking device divided into two sub-regions made of the drilled GRIN PC and the Si-inclusion filled GRIN PC. (**c**) The relative wave speed 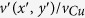

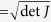
of the S0 Lamb wave mode at 100 kHz as a function of the inclusion radius 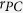 for two different inclusion materials (air and silicon). (**d**) The arrangement of air holes and Si inclusions of varying diameters 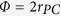 (unit: mm) to realize an elastic annular cloak encircling a hole of radius 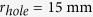. The circles above and below the dotted line denote air holes and Si inclusions, respectively.

To make a cloaking device that satisfies 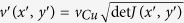, two types of GRIN PCs (Gradient-Refractive-Indexed Phononic Crystals) were used to build sub-regions 1 and 2. Figure 2a shows square unit cells filled with air and square unit cells filled with Si inclusions that are used to make a GRIN PC for sub-regions 1 and 2, respectively. As the wave speed in the Si plate (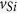 = 7778.3 m/sec) is faster than the wave speed in the Cu plate (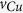 = 3759.4 m/s) for the S0 wave mode propagating at 100 kHz, Si inclusions must be used for sub-region 2. The distribution of the unit cells having different inclusion types and sizes is plotted in Fig. 2b. The wave speed 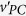 as a function of the inclusion radius 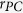 is given in Fig. 2c. The wave speed in the PC, denoted by 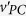, is calculated as the average value of the two wave speeds along two directions, 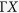
and 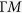 (shown in Fig. 2a), in accordance with prior research calculations^27^. Although the desired largest value of 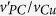 required at some locations in sub-region 2 is 5, the largest value of 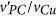 that can be realized with Si inclusions is approximately 1.5, as shown in Fig. 2c. Therefore, realizing the zone of 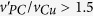 by using the Si-inclusion PC is not possible. However, such high velocities are required only in a very limited zone, so the performance of the Si- and air-filled PC based metamaterial cloaks will not be significantly affected, even if the desired high velocities are not exactly achieved. This issue is carefully examined in the Supplementary Information section. Figure 2d shows the actual arrangements of air holes and Si inclusions of varying radii of the GRIN PC to make the metamaterial elastic cloaks.

At this point, it is worth emphasizing the difference between the add-on cloak and the embedded cloak. First, the embedded cloak is made in the Cu plate having a hole of radius 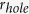 = 15 mm by drilling the plate with a 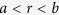 size hole, and then filling the drilled-out holes with air (for sub-region 1) or Si (for sub-region 2). However, the add-on cloak was made as a separate device and is attached (bonded for the experiment) on the Cu plate with a hole of radius 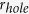 = 15 mm. The thickness of the add-on metamaterial cloak has a varying profile to produce no slope discontinuity at 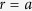 and 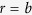. In this way, the wave reflection, especially at *r = b,* will be minimized due to the absence of an abrupt impedance mismatch. In the present case, a Bezier curve was used to define the thickness profile (see Fig. 1a).

Even with the smoothly varying thickness profile, when employing the add-on cloaking device, the add-on cloaking device and the original Cu plate together form a new composite structure covering the annular region of 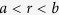. Therefore, the desired velocity field given by Eq. (9) and Eq. (10) may not be achievable because the cloak is added onto the plate. Nevertheless, the add-on cloak can still function as a cloaking device as the incident symmetric wave propagates over the total thickness of the cloak and plate assembly. This will also be demonstrated and confirmed in the experimental results.

### Experiments and Findings

**Figure 3:**
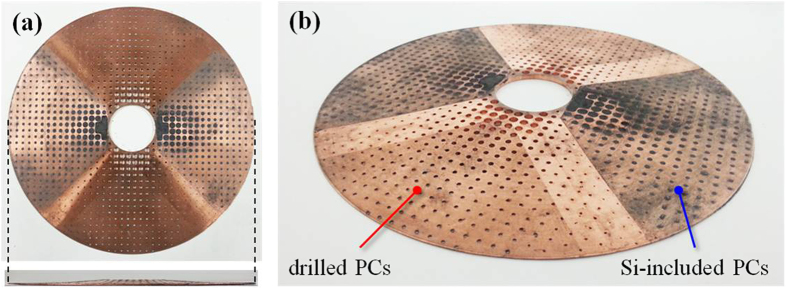
Photos of the add-on cloaking device used as a stress bandage cloak. (**a**) Top view. (**b)** Side view, with illustrations of the drilled and the Si-included PC areas. The thickness of the cloak is varied according to the Bezier curve.

**Figure 4:**
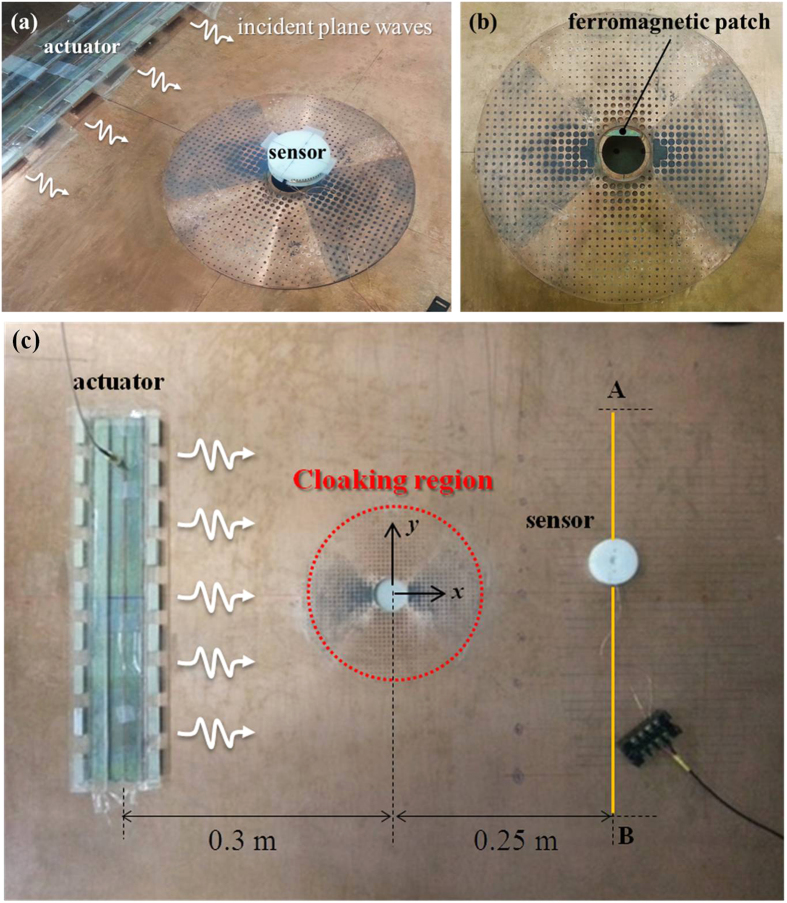
Experimental setup in a 3-mm Cu plate of a circular hole (*r*_*hole*_ = 15 mm). (**a**) Photo of the add-on cloak device bonded onto the plate. The actuator made of a giant magnetostrictive transducer excites a plane symmetric Lamb wave and the sensor made of a magnetostrictive transducer measures the Lamb wave. (**b**) Zoomed photo of the installed add-on cloak. For the photo, the magnetic circuit of the sensing unit (magnetostrictive transducer) is removed, and only a thin magnetostrictive patch shaped in a circle segment is shown. (**c**) Photo of the experimental setup in a 3-mm Cu plate with an embedded cloak around a circular hole of 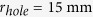. The actuator and sensor are also magnetostrictive patch transducers.

Figure 3 shows the photo of the add-on cloaking device fabricated according to the design shown in Fig. 1a. It is made of a thickness-varying annular Cu plate that is hole-drilled. The diameters of air holes and Si inclusions have been given in Fig. 2d. The Si inclusions are formed by sintering a mixture of Si and a binder and then compressing into holes. The add-on cloaking device used for the experiments is bonded onto a Cu plate having a hole of radius 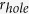, as shown in Fig. 4a,b. For comparison, a wave experiment with a cloak embedded in the Cu plate, shown in Fig. 4c, was also performed.

The setups shown in Fig. 4a,c were used to perform the experiments for the add-on cloaking device and the embedded clocking device, respectively. A magnetostrictive transducer^23^ is used as an actuator for both experiments. It operates through the magnetostrictive effect or the Joule effect^28^,^29^. The actuator fabricated for the experiments may be viewed as a modified giant version of a Magnetostrictive Patch Transducer (MPT) using a planar solenoid array^24^,^25^,^26^. A similar giant MPT was used in an earlier work^30^. When a Gabor (modulated Gaussian) pulse centered at 100 kHz amplified by a voltage amplifier is sent into the solenoid, the magnetostrictive patch bonded on the plate deforms at the rate of the excitation frequency and thereby generates the S0 Lamb wave in the plate. The desired plane S0 wave requires that the transducer should be much longer in its vertical dimension than in its horizontal dimension. The actuating MPT has several sets of permanent magnets to provide the magnetostrictive patch with the uniform biasing static magnetic field required for its operation.

The sensing transducer in Fig. 4c is the original version of the MPT using a circular magnetostrictive patch and a figure-of-eight coil^24^,^25^. The magnetic circuit is formed with permanent magnets, and the figure-of-eight coil is simply placed over the circular patch. The sensor shown in Fig. 4c was used to measure the stress distribution along the 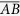 line (not shown in Fig. 4a) for the experiments with the add-on and embedded cloaks. The sensor shown on the top of the metamaterial cloak in Fig. 4a was used to measure the maximum stress along the circular boundary of the hole in the plate. Because the gap between the boundary of the hole and the inner radius of the add-on cloak is only 5 mm, we made some modifications from similar MPTs used previously^24^,^25^. Thus, a circular-segment magnetostrictive patch was employed instead of a circular magnetostrictive patch. Figure 4b shows the circular-segment patch made of nickel bonded onto the Cu plate. Additionally, the magnetic circuit of the permanent magnets and the figure-of-eight coil cannot be placed directly onto the Cu plate due to the finite thickness (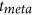) of the add-on cloak at 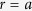, but it could be placed at an offset of 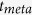
** = **3 mm. For all the measurements, 1000 samples were averaged.

Then, the performance of the add-on cloaking device was investigated against the performance of the embedded cloaking device. The effectiveness of the add-on cloaking device was examined in terms of its capability to recover from the perturbed far-field wave field by the presence of a hole. In other words, the stress distribution along 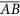 was analyzed. Finally, the degree of mitigation in stress concentration by the application of the add-on cloaking device was also investigated.

**Figure 5:**
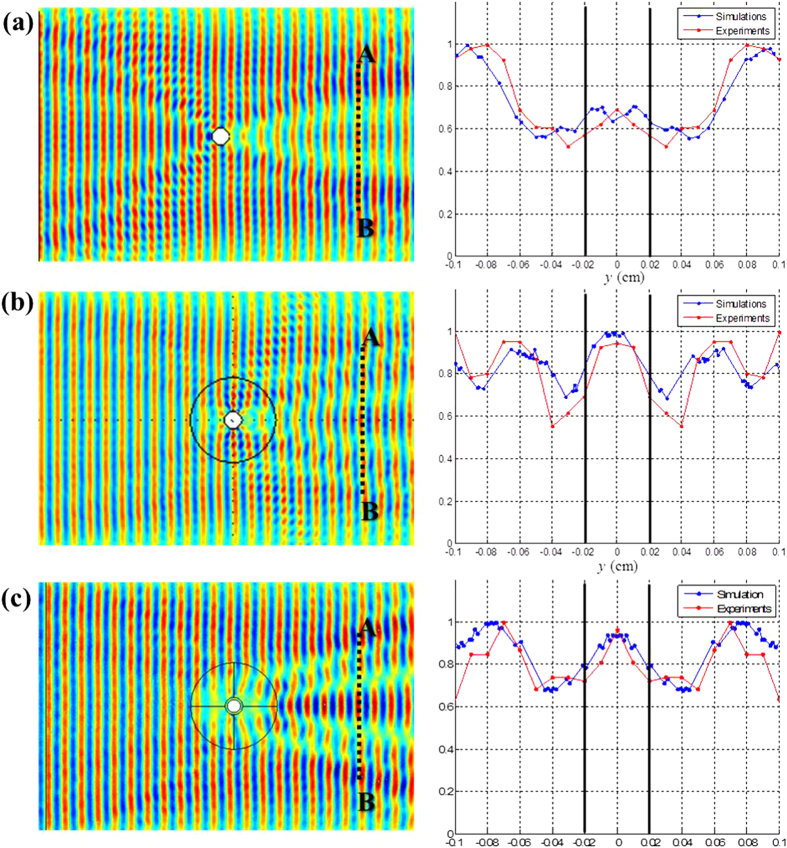
The normal stress (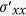) field in the Cu plate with and without the cloaking device for the horizontal incidence of the S0 wave at 100 kHz and the normalized normal stress distribution along the 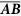 line. (See Fig. 1a for the definition of the coordinate axes). (**a**) No cloaking. (**b**) The use of the embedded cloaking device. (**c**) The use of the add-on cloaking device. The signals were measured at every 10 cm along the 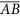 line.

Figure 5 compares the wave field of the dominant stress component 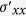 in the plate. Also, its distribution along the 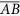 line was plotted (ranging from 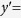 −10 cm to 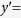 10 cm for a fixed valued of 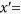
25 cm). First, the results of the wave simulation with the embedded cloaking device were compared with those of the wave simulation without the device. When no cloaking device is installed, the normal stress 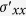, especially at the center of the 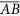 line (i.e., at *y* = 0), is significantly perturbed due to the presence of a hole in the Cu plate, and at some points along the 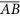 line, the stress is reduced to approximately 70% of the maximum stress level. The maximum level virtually corresponds to the stress level in a plate without any hole that is subjected to the plane wave incidence of the symmetric Lamb wave mode. However, the stress level is recovered to almost 95% of the maximum value when the embedded cloaking device is installed. Although the recovered stress field is not fully uniform along 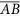, the stress distribution over 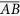 in the plate with the cloak is considerably less disturbed than that in the plate without the cloak. This confirms that more uniformly distributed wave signals could be obtained if the cloak is used. The developed cloak is not perfect, but it is observed that it works almost as well as earlier elastic cloaks that were considered for other applications^4^,^5^,^6^.

Next, we compared the stress distribution along 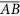 in the plates with the embedded and add-on cloaks as shown in the plots in Fig. 5b,c, respectively. The comparison reveals that there is no significant difference in the embedded and add-on cloaks in terms of cloaking performance. This supports the proposition that the functioning of the add-on type cloak is almost equal to that of the embedded cloak in minimizing the perturbation of the stress field after the damage hole is created in the region. Because the add-on type cloak can be installed on a damaged plate without machining the damaged plate, this finding could be crucial for further developments in engineering applications of metamaterial cloaks. As a means to show the effectiveness of the proposed add-on cloak, we calculated the scattering cross section 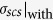
in the holey plate with the add-on cloak and also the scattering cross section 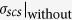 in the holey plate without the cloak. The wave fields used for the calculations are shown in Fig. 5c,a. The ratio of 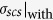
to 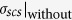 was 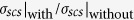 = 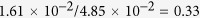; the add-on cloak reduced the scattering cross section by 67%.

We investigated the effectiveness of the add-on cloak device in the reduction of the stress concentration caused by the presence of a hole in the plate. It may be noted that if a cloak is ideal, there would be no stress concentration around the hole, and hence our discussion is based on the fact that no ideal cloak can be engineered in practice. In the case of the plane S0 Lamb wave incidence, the maximum stress (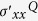) occurs at *Q* (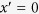, 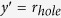 = 15 mm), where *Q* denotes the known location of the maximum stress. Therefore, 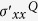 is mainly examined. First, the magnitudes of 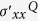 in the Cu plate with a hole before and after applying the add-on cloak are compared through numerical wave simulations. The simulation reveals the following expression:


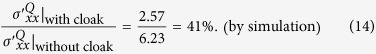


**Figure 6:**
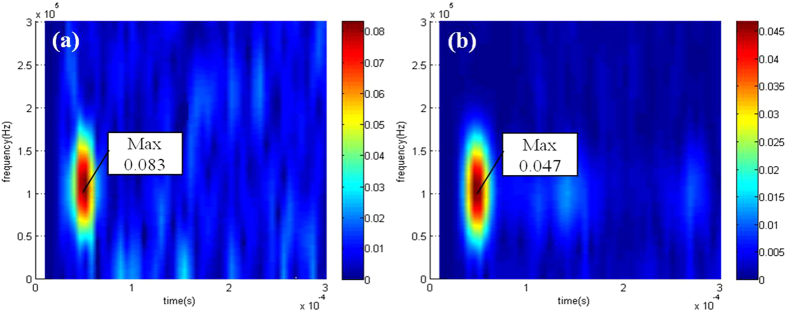
The short-time Fourier transform of the measured signal by the sensing MPT at *Q* in the Cu plate with a hole (**a**) before and (**b**) after the add-on metamaterial cloak was applied. The values of the arbitrary unit denote the magnitude of the measured stress signal at 100 kHz.

The result (14) implies that the reduction in the maximum stress is approximately 59%. This appears to be a significant reduction in the maximum stress. To experimentally find the value of 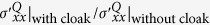, the wave signals at *Q* were measured by using the magnetostrictive transducers (MPTs) elaborated in the earlier section. Figure 6 shows the short-time Fourier transform of the measured data. From the result in Fig. 6, the stress value for the frequency component at 100 kHz is found to be as follows:


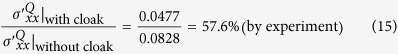


The result in Eq. (15) shows that the maximum stress is reduced in the experiments by 42.4%. The difference between the experimental and the simulation results is due to several reasons, including errors in the fabrication of the cloak device. The main reason for this is the difficulty in making exact stress measurements at *Q* because the magnetostrictive patches of the measured MPT occupy some finite areas while the stress around *Q* varies rapidly.

Finally, investigating the amount by which the stress concentration can be mitigated if a “pure annular solid” Cu plate with the same geometry as the add-on cloak is simply installed over the plate with a hole instead of using the add-on metamaterial cloak, is important. For this study, only simulations were performed. The detailed stress field is not included here, but the following conclusions were drawn from the analysis of the simulation results. First, stress reduction through a simple installation of an annular Cu plate was only 24%, which is quite small when compared with that of an add-on cloak (59%). As expected, the stress distribution along the 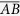 line was not as uniform as that obtained with the use of the proposed add-on metamaterial cloak.

## Conclusions

A new concept of an add-on elastic metamaterial cloak for plate applications was suggested. It was tested as a potential device called a “stress (alleviating) bandage” to be installed over a damaged region of a thin plate. It was devised to mitigate the stress concentration around a hole or any other damage and also to recover the perturbed far wave field back to the original unperturbed wave field without requiring a hole. This study presented the design of such a device and its fabrication by GRIN PCs. Additionally, wave experiments in a plate that was subjected to the incidence of a 100 kHz symmetric plane Lamb wave were performed.

From this study, the following conclusions can be made. The cloaking performance of the proposed add-on elastic metamaterial cloak was found to be comparable to that of an embedded cloak, suggesting that by the presence of a hole in the plate, the add-on cloak can be used to recover the perturbed wave field back to the wave field before the creation of the hole. In addition, the stress concentration due to the application of the add-on type was significantly mitigated by almost 59% in the simulation, and 42% in the experiments. The suggested conformal mapping (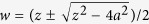) was found effective in designing an elastic metamaterial cloak by isotropic media because the 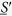 and 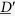 terms breaking the form-invariance of the transformation method may be ignored. We believe that the experimental results with an add-on metamaterial plate clock could open up new possibilities for using it as an onsite emergency stress-relieving device or an onsite stress bandage in various applications. Despite the promising results that were obtained for the proposed add-on metamaterial cloak, there are still several issues to be resolved. For instance, considerably high frequencies were used to facilitate the experiments in which the metamaterial cloak could be realized by GRIN PCs. In the case of low-frequency excitations, other methods should be developed to fabricate the add-on cloaks. Add-on metamaterials that work over a broad frequency range should also be designed. The effects of the various approximations made in the course of fabricating and testing the present add-on elastic metamaterial cloak should be further analyzed, both quantitatively and qualitatively. The design of better mapping functions should also be investigated. Because the present add-on cloak device works only for unidirectional stress wave incidence, further research should be conducted to design isotropic add-on cloaks.

## Additional Information

**How to cite this article**: Lee, M. K. and Kim, Y. Y. Add-on unidirectional elastic metamaterial plate cloak. *Sci. Rep.* **6**, 20731; doi: 10.1038/srep20731 (2016).

## Supplementary Material

Supplementary Information
