# Supplementary material for: Add-on unidirectional elastic metamaterial plate cloak
Source: Sci Rep. 2016 Feb 10;6:20731. doi: 10.1038/srep20731 (PMC4748288; doi:10.1038/srep20731)
Supplement: Supplementary Information [file srep20731-s1.pdf]

Add-on unidirectional elastic metamaterial plate cloak

Min Kyung Lee[*a] and Yoon Young Kim[**a,b]

[a]*Department of Mechanical and Aerospace Engineering, Seoul National University, 1 Gwanak-ro, Gwanak-gu, Seoul, 151-742, Korea*

[b]*Institute of Advanced Machine and Design, Seoul National University, 599 Gwanak-ro, Gwanak-gu, Seoul, 151-744, Korea*

The transformation elasticity[3] using the conformal mapping function given by Eq. (2) was used to design an add-on metamaterial plate cloak. More detailed analysis and the use of an approximate field equation of Eq. (9) along with the assumptions of $\rho' \simeq \rho,\ \ D'_{pqr} = S'_{qrp} \approx 0$ will be justified. Because we are mainly concerned with the lowest symmetric Lamb waves, we will consider two-dimensional wave propagation problems.

Conformal mapping functions were used earlier to design cloaks with isotropic material behavior for use in acoustic and electromagnetic problems, and in these cases, form-invariance was preserved. One of the main motivations to use a

[*] Current Address: *Samsung Advanced Institute of Technology, 130 Samsung-ro, Yeongtong-gu, Suwon, Gyeonggi-do, 443-803, Korea*

[**] Corresponding Author, yykim@snu.ac.kr

conformal mapping function for the present elasticity problem is to ignore the terms breaking the form-variance in order to engineer the cloaking device. This approximation may be justified as shall be shown below.

We will begin with a well-known elastodynamic equation stated in the original Cartesian coordinates ($x_1 = x, x_2 = y$) as

$$\nabla \cdot \left(\underline{C} \nabla \bar{u} \right) = -\omega^2 \rho \bar{u} \qquad \text{(S1)}$$

where the displacement vector $\bar{u}$ is assumed to be time-harmonic at the angular frequency of ω as

$$\text{dispacement vector} = \bar{u} \exp(i\omega t) . \qquad \text{(S2)}$$

In Eq. (1), $\underline{C}$ and ρ denote the stiffness tensor and density. For the case with isotropic media, the components of $\underline{C}$ can be expressed in terms of the Lame constants (λ , μ) as

$$C_{ijkl} = \lambda \delta_{ij} \delta_{kl} + \mu(\delta_{ik} \delta_{jl} + \delta_{il} \delta_{jk}) \qquad \text{(S3)}$$

where δ_{ij} is the Kronecker delta.

If the transformation from the unprimed (*x*,*y*) coordinate system to the primed (x', y') coordinate system is written as

$$x' = f(x, y), \quad y' = g(x, y) \qquad \text{(S4)}$$

The Jacobian matrix J comes

$$J = \begin{bmatrix} \dfrac{\partial x'}{\partial x} & \dfrac{\partial x'}{\partial y} \\ \dfrac{\partial y'}{\partial x} & \dfrac{\partial y'}{\partial y} \end{bmatrix} \tag{S5}$$

By the transformation in Eq. (S4), the displacement vector $\bar{u}(x, y)$ is mapped to

$$\bar{u}'(x', y') = J^{-T}(x, y)\bar{u}(x, y) \tag{S6}$$

and the governing elastodynamic equation becomes, in the primed coordiates,

$$\nabla' \cdot \left(\underline{C}' \nabla' \bar{u}' + \underline{S}' \bar{u}' \right) = \underline{D}' \nabla' \bar{u}' - \omega^2 \underline{\rho}' \bar{u}' \tag{S7}$$

The primed quantities in Eq. (S7) denote the quantities in the primed coordinate system. The density tensor $\underline{\rho}'$, the transformed stiffness tensor $\underline{C}'$, and the coupling tensors $\underline{S}'$ and $\underline{D}'$ defined in the primed coordinates can be explicitly written as[2]

$$\rho'_{pq} = \frac{\rho}{\det J} \frac{\partial x'_p}{\partial x_i} \frac{\partial x'_q}{\partial x_i} + \frac{1}{\det J} \frac{1}{(-i\omega)^2} \frac{\partial^2 x'_p}{\partial x_i \partial x_j} C_{ijkl} \frac{\partial^2 x'_q}{\partial x_k \partial x_l} \tag{S8}$$

$$C'_{pqrs} = \frac{1}{\det J} \frac{\partial x'_p}{\partial x_i} \frac{\partial x'_q}{\partial x_j} C_{ijkl} \frac{\partial x'_r}{\partial x_k} \frac{\partial x'_s}{\partial x_l} \tag{S9}$$

$$S'_{pqr} = \frac{1}{\det J} \frac{\partial x'_p}{\partial x_i} \frac{\partial x'_q}{\partial x_j} C_{ijkl} \frac{\partial^2 x'_r}{\partial x_k \partial x_l} = S'_{qpr} \tag{S10}$$

$$D'_{pqr} = \frac{1}{\det J}\frac{\partial^2 x'_p}{\partial x_i \partial x_j} C_{ijkl} \frac{\partial x'_q}{\partial x_k}\frac{\partial x'_r}{\partial x_l} = S'_{qrp}$$

(S11)

Note that unlike in Eq. (S1), the new $\underline{S}'$ and $\underline{D}'$ quantities appear in Eq. (S7). The existence of these terms complicates the use of the transformation method for engineering applications because these terms cannot be realized with ordinary materials alone. Therefore, pentamode metamaterials [3, 37, 38] have been suggested in order to diminish the influence of the $\underline{S}'$ and $\underline{D}'$ terms. Since it is not easy to make such special materials, our approach is to use a transformation that makes the terms involving $\underline{S}'$ and $\underline{D}'$ as small as possible so that they can be ignored for the actual realization of any metamaterial device. In other words, it would be possible to approximate Eq. (S7) as the following equation for a certain mapping function:

$$\nabla' \cdot \left(\underline{C}' \nabla' \bar{u}' \right) \approx -\omega^2 \rho' \bar{u}' \qquad \text{(S12)}$$

We argue that if a conformal mapping function is used for the transformation, the use of Eq. (S12) can be justified for engineering applications. This approximation provided by this equation may be compared to the result of the form-preserving transformation method used for electromagnetics and acoustics.

In the case of the two-dimensional analysis, a conformal mapping or transformation[R3-R4] is known to preserve the original angle between two arbitrary lines after the transformation. Therefore, it can be conveniently expressed in terms of a complex function. Earlier uses of conformal mapping were found in Refs. [2, 13], where invisibility electromagnetic and acoustic cloaks were designed. Conformal mapping is also known to preserve isotropy in the density after the transformation is applied in acoustics. The use of conformal mapping functions for elastodynamics can be found in Refs. [15-18], but their goal was not to approximate Eq. (S7) as in Eq. (S12). The elastic wave control by using the conformal transformation was studied in Refs. [19-22]. In these studies, they applied the conformal transformation locally to infinitesimal parts of solid media so that an insignificant influence of the extra $\underline{S}'$ and $\underline{D}'$ terms on the transformed elastodynamic equation would not be a concern. Here, we select a specific mapping function for an elastic cloaking device intended for use toward stress concentration mitigation. In doing so, we must investigate the extent to which the exact transformed equation (S7) is affected if the approximation of Eq. (S12) is used.

Let us now investigate what happens to the terms involving $\underline{S}'$ and $\underline{D}'$ in Eq. (S7) if the conformal mapping function is used. If Eq. (S4) represents a conformal

transformation, the well-known Cauchy-Riemann condition must be satisfied:

$$\frac{\partial x'}{\partial x}=\frac{\partial y'}{\partial y},\qquad \frac{\partial x'}{\partial y}=-\frac{\partial y'}{\partial x} \tag{S13a,b}$$

with

$$J=\begin{bmatrix}\dfrac{\partial x'}{\partial x} & \dfrac{\partial x'}{\partial y}\\ \dfrac{\partial y'}{\partial x} & \dfrac{\partial y'}{\partial y}\end{bmatrix}\equiv\begin{bmatrix}\alpha & \beta\\ -\beta & \alpha\end{bmatrix},\qquad \det J=\alpha^2+\beta^2 \tag{S14a,b}$$

Using Eq. (S13),

$$\frac{\partial^2 x'}{\partial x^2}=\frac{\partial^2 y'}{\partial x\partial y},\quad \frac{\partial^2 x'}{\partial x\partial y}=\frac{\partial^2 y'}{\partial y^2},\quad \frac{\partial^2 x'}{\partial x\partial y}=-\frac{\partial^2 y'}{\partial x^2},\quad \frac{\partial^2 x'}{\partial y^2}=-\frac{\partial^2 y'}{\partial x\partial y} \tag{S15a,b,c,d}$$

Substituting Eqs. (S13-15) into Eq. (S8) with $(p,q)=(xx)$ yields

$$\begin{aligned}
\rho'_{xx} &= \rho-\frac{1}{\det J}\frac{1}{\omega^2}\left((\lambda+2\mu)\left(\left(\frac{\partial^2 y'}{\partial x\partial y}\right)^2+\left(\frac{\partial^2 y'}{\partial x\partial y}\right)^2\right)-2\lambda\left(\frac{\partial^2 y'}{\partial x\partial y}\right)^2+4\mu\left(\frac{\partial^2 x'}{\partial x\partial y}\right)^2\right)\\
&= \rho-\frac{1}{\det J}\frac{1}{\omega^2}\left(2(\lambda+2\mu)\left(\frac{\partial^2 y'}{\partial x\partial y}\right)^2-2\lambda\left(\frac{\partial^2 y'}{\partial x\partial y}\right)^2+4\mu\left(\frac{\partial^2 x'}{\partial x\partial y}\right)^2\right)\\
&= \rho-\frac{1}{\det J}\frac{1}{\omega^2}\left(4\mu\left(\frac{\partial^2 y'}{\partial x\partial y}\right)^2+4\mu\left(\frac{\partial^2 x'}{\partial x\partial y}\right)^2\right)\\
&= \rho-\frac{4\mu}{\det J}\frac{1}{\omega^2}\left(\left(\frac{\partial^2 x'}{\partial x\partial y}\right)^2+\left(\frac{\partial^2 y'}{\partial x\partial y}\right)^2\right)
\end{aligned} \tag{S16}$$

Note that the density term ρ'_{xx} in the transformed space involves only the shear modulus μ (not λ) if a conformal mapping is employed. Repeating for other

terms, such as ρ'_{yy} and ρ'_{xy}, we find

$$\underline{\rho'} = \begin{bmatrix} \rho'_{xx} & \rho'_{xy} \\ \rho'_{yx} & \rho'_{yy} \end{bmatrix} = \begin{bmatrix} \rho'_{xx} & 0 \\ 0 & \rho'_{xx} \end{bmatrix} = \rho'_{xx} \mathbf{1} \tag{S17}$$

where $\mathbf{1}$ is a 2×2 identity tensor. Since the density in the transformed space is written as an identity tensor, the material composing the elastic cloak is isotropic in density, which is desirable for an actual cloak.

This time, the stiffness tensor will be examined in the transformation space. When a conformal transformation was used, the following results were obtained:

$$\begin{aligned} C'_{1111} &= \frac{1}{\det J}\left[C_{1111}\left(\alpha^4+\beta^4\right)+2C_{1122}\alpha^2\beta^2+4C_{1212}\alpha^2\beta^2\right] \\ &= \frac{1}{\det J}\left[(\lambda+2\mu)\left(\alpha^4+\beta^4\right)+2\lambda\alpha^2\beta^2+4\mu\alpha^2\beta^2\right] \\ &= \frac{1}{\det J}\left[\lambda\left(\alpha^4+2\alpha^2\beta^2+\beta^4\right)+2\mu\left(\alpha^4+2\alpha^2\beta^2+\beta^4\right)\right] \\ &= \frac{1}{\det J}(\lambda+2\mu)\left(\alpha^2+\beta^2\right)^2 = \frac{1}{\det J}(\lambda+2\mu)(\det J)^2 \\ &= \det J\cdot(\lambda+2\mu) = \det J\cdot C_{1111} = C'_{2222}, \end{aligned} \tag{S18a}$$

$$\begin{aligned} C'_{1122} &= \frac{1}{\det J}\left[2(\lambda+2\mu)\alpha^2\beta^2+\lambda\left(\alpha^4+\beta^4\right)-4\mu\alpha^2\beta^2\right] \\ &= \frac{1}{\det J}\left[2\lambda\alpha^2\beta^2+\lambda\left(\alpha^4+\beta^4\right)\right] = \frac{1}{\det J}\lambda\left(\alpha^4+2\alpha^2\beta^2+\beta^4\right) \\ &= \frac{1}{\det J}\lambda\cdot(\det J)^2 = \det J\cdot\lambda = \det J\cdot C_{1122}, \end{aligned} \tag{S18b}$$

$$\begin{aligned} C'_{1212} &= \frac{1}{\det J}\left[C_{1111}\cdot 2\alpha^2\beta^2 - C_{1122}\cdot 2\alpha^2\beta^2 + C_{1212}\left(\alpha^4 - 2\alpha^2\beta^2 + \beta^4\right)\right] \\ &= \frac{1}{\det J}\left[2\left(\lambda+2\mu\right)\alpha^2\beta^2 - 2\lambda\alpha^2\beta^2 + \mu\left(\alpha^4 - 2\alpha^2\beta^2 + \beta^4\right)\right] \\ &= \det J\cdot\mu = \det J\cdot C_{1212}\ \ (= C'_{1212} = C'_{1221} = C'_{2112} = C'_{2121}), \end{aligned} \tag{S18c}$$

$$\begin{aligned} C'_{1112} &= \frac{1}{\det J}\left[C_{1111}\left\{\alpha^3\cdot(-\beta)+\beta^3\alpha\right\} + C_{1122}\left\{\alpha^2\beta\alpha+\beta^2\alpha\cdot(-\beta)\right\} + C_{1212}\left(2\alpha^3\beta - 2\alpha\beta^3\right)\right] \\ &= \frac{1}{\det J}\left[-\left(\lambda+2\mu\right)\alpha\beta\left(\alpha^2-\beta^2\right) + \lambda\alpha\beta\left(\alpha^2-\beta^2\right) + 2\mu\alpha\beta\left(\alpha^2-\beta^2\right)\right] = 0 \end{aligned} \tag{S18d}$$

The result in Eqs. (S18a-d) derived for the stiffness tensor components in the transformed space implies that

$$\underline{C}' = \det J\cdot\underline{C} \tag{S19}$$

The relation given by Eq. (S19), which is the same Eq. (3), is very useful because the stiffness tensor $\underline{C}'$ can be also isotropic with a scale factor of $\det J$ if the base material in the original space is isotropic.

Next, the components of $\underline{S}'$ will be examined:

$$\begin{aligned} S'_{111} &= \frac{1}{\det J}\left[(\lambda+2\mu)\left(\alpha^2\frac{\partial^2 x'}{\partial x^2} + \beta^2\frac{\partial^2 x'}{\partial y^2}\right) + \lambda\left(\alpha^2\frac{\partial^2 x'}{\partial y^2} + \beta^2\frac{\partial^2 x'}{\partial x^2}\right) + 4\mu\alpha\beta\frac{\partial^2 x'}{\partial x\partial y}\right] \\ &= \frac{1}{\det J}\left[(\lambda+2\mu)\left(\alpha^2-\beta^2\right)\frac{\partial^2 y'}{\partial x\partial y} - \lambda\left(\alpha^2-\beta^2\right)\frac{\partial^2 y'}{\partial x\partial y} + 4\mu\alpha\beta\frac{\partial^2 x'}{\partial x\partial y}\right] \\ &= \frac{2\mu}{\det J}\left[\left(\alpha^2-\beta^2\right)\frac{\partial^2 x'}{\partial x^2} + 2\alpha\beta\frac{\partial^2 y'}{\partial y^2}\right] \end{aligned} \tag{S20a}$$

$$S'_{112} = \frac{2\mu}{\det J}\left[2\alpha\beta\frac{\partial^2 x'}{\partial x^2} - \left(\alpha^2 - \beta^2\right)\frac{\partial^2 y'}{\partial y^2}\right] \tag{S20b}$$

The remaining terms in $\underline{S}'$ can be expressed in terms of S'_{111} and S'_{112} as

$$S'_{111} = S'_{122} = S'_{212} = -S'_{221} \tag{S20c}$$

$$S'_{112} = -S'_{121} = -S'_{211} = -S'_{222} \tag{S20d}$$

The examination of Eqs. (S20) shows that among two material stiffness properties (λ and μ), S'_{ijk} involves only the μ term (shear modulus) when the conformal mapping is used. This means that the S'_{ijk} terms due to the conformal transformation could be small relative to the S'_{ijk} terms of a non-conformal transformation. Therefore, the $\underline{S}'$ (and $\underline{D}'$) terms may be omitted in Eq. (S7) if a conformal mapping function is used. This observation will be investigated through numerical simulations with the selected conformal mapping function, Eq. (2).

Since $D'_{pqr} = S'_{qrp}$, it is sufficient to examine the influence of the terms involving $\underline{S}'$ in Eq. (S7). Note that the terms $\underline{C}'\nabla'\bar{u}' + \underline{S}'\bar{u}'$ appearing on the left-hand side of Eq. (S7) originate[3] from $\sigma' = \underline{C}'\nabla' u' + \underline{S}' u' = \underline{C}'\varepsilon' + \underline{S}' u'$ where σ' and ε' denote the stress tensor in the transformed space. The relative contribution of $\underline{S}' u'$ to σ' is estimated by comparing it to the contribution of

$\underline{C}'\varepsilon'$ to σ' by introducing the following measures:

$$R_{xx} = \left[\frac{S'u'}{C'\nabla'u'}\right]_{xx} = \frac{S'_{111}u_x' + S'_{112}u_y'}{C'_{1111}\cdot\varepsilon'_{xx} + C'_{1112}\cdot\varepsilon'_{xy} + C'_{1121}\cdot\varepsilon'_{yx} + C'_{1122}\cdot\varepsilon'_{yy}} \quad \text{(S21a)}$$

$$R_{xy} = \left[\frac{S'u'}{C'\nabla'u'}\right]_{xy} = \frac{S'_{121}u_x' + S'_{122}u_y'}{C'_{1211}\cdot\varepsilon'_{xx} + C'_{1212}\cdot\varepsilon'_{xy} + C'_{1221}\cdot\varepsilon'_{yx} + C'_{1222}\cdot\varepsilon'_{yy}} \quad \text{(S21b)}$$

$$R_{yy} = \left[\frac{S'u'}{C'\nabla'u'}\right]_{yy} = \frac{S'_{221}u_x' + S'_{222}u_y'}{C'_{2211}\cdot\varepsilon'_{xx} + C'_{2212}\cdot\varepsilon'_{xy} + C'_{2221}\cdot\varepsilon'_{yx} + C'_{2222}\cdot\varepsilon'_{yy}} \quad \text{(S21c)}$$

where u_x' and u_y' denote the displacement components in the transformed space along the x' and y' axes. These measures are affected not only by the $\underline{S}'$ and $\underline{C}'$ terms, but also by the selected transformations, displacements, and strain fields. Therefore, they must be verified for specific applications. However, if the values of the measures are much smaller than unity, ignoring the $\underline{S}'$ and $\underline{C}'$ terms is justified. In this case, the cloaking device can be made of a compound of isotropic materials.

Two transformations, the conformal mapping in Eq. (2) and another non-conformal mapping, will be considered to demonstrate the advantage of the conformal mapping function over the non-conformal mapping function. First of all, consider the following non-conformal mapping function that is expressed in the polar coordinates (r,θ) and (r',θ'):

$$r' = a + r(b-a)/b, \ \ \theta' = \theta \qquad \text{(S22)}$$

where a and b are the inside and outside radii of a cloak. The conformal mapping function given by Eq. (2) is restated here for the sake of convenience:

$$w = \frac{1}{2}\left(z \pm \sqrt{z^2 - 4a^2}\right) \qquad \text{(S23a)}$$

with

$$z = x + iy \ \ \text{and} \ \ w = x' + iy' \qquad \text{(S23b)}$$

The transformations given by Eqs. (S22, S23) are illustrated in Fig. S1. There are several differences between the two transformations. First, the annular region between $r=b$ and $r=a$ is affected by Eq. (S22) while the entire space is affected by Eq. (S23). However, the non-conformal mapping requires an anisotropic medium in the cloak region, which is extremely difficult to realize. On the other hand, the region outside of $r=b$ by conformal transformation is distorted, but the degree of the distortion is quite small, and as a result, the original homogeneous isotropic material can be used to fill the outside region. Obviously, the advantage of using a conformal mapping function is that the cloaked region can be realized with an isotropic medium.

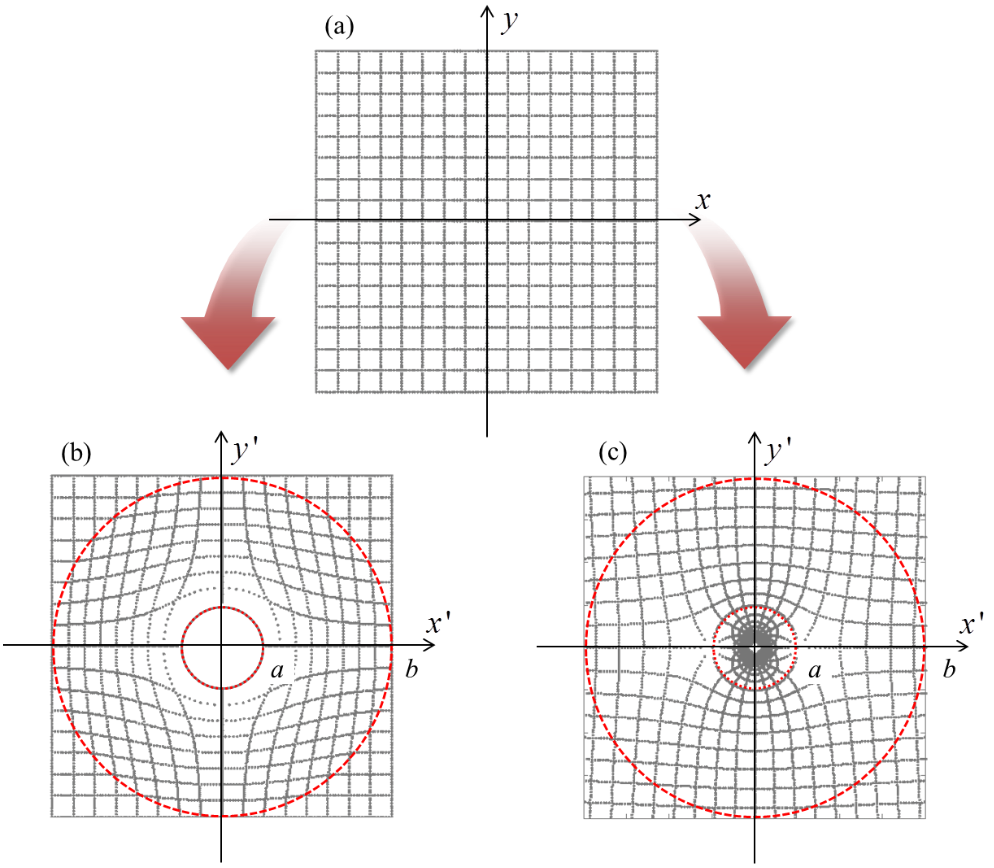

Figure S1. (a) An original space. (b) The space transformed by the non-conformal transformation (S22) and (c) the space transformed by the conformal transformation (S23) for elastic cloaking with an incident wave along the x' direction.

Figure S2 shows wave simulations in a plate with an embedded cloaking device covering the region of $a < r < b$ (a= 2 cm, b= 10, plate thickness = 3 mm). All wave simulations in this work were performed using COMSOL Multiphysics. The material properties of the annular cloak were calculated with the results

derived earlier, and the original space is assumed to be filled with Al. For the simulations, the incident wave was the lowest symmetric Lamb-type symmetric (denoted by S0) time-harmonic wave centered at 150 kHz. (Almost similar results were obtained at 100 kHz for other media such as Cu.) It is a plane wave that propagates along the horizontal axis. The results of the simulation with and without ignoring the $\underline{S}'$ and $\underline{C}'$ terms are compared for both transformations in Fig. S2. When the non-conformal transformation was used, the wave fronts become noticeably perturbed if the $\underline{S}'$ and $\underline{C}'$ terms were ignored. On the other hand, when conformal mapping was used, they were relatively unaffected even if the $\underline{S}'$ and $\underline{C}'$ terms were ignored. The displacement in the x' direction is plotted in Fig. S2 along $\overline{AB}$ with and without the $\underline{S}'$ and $\underline{C}'$ terms. It clearly demonstrates that ignoring the $\underline{S}'$ and $\underline{C}'$ terms has considerably different effects, depending on the type of transformation functions that are employed. Although there are some peaks in the displacement plots near the outer radius of the circular cloaking device, the displacement distribution due to conformal mapping is more even than that produced through non-conformal mapping. Therefore, one can conclude for the case with the conformal mapping of Eq. (S23) that ignoring the $\underline{S}'$ and $\underline{C}'$ terms does not significantly alter the wave field.

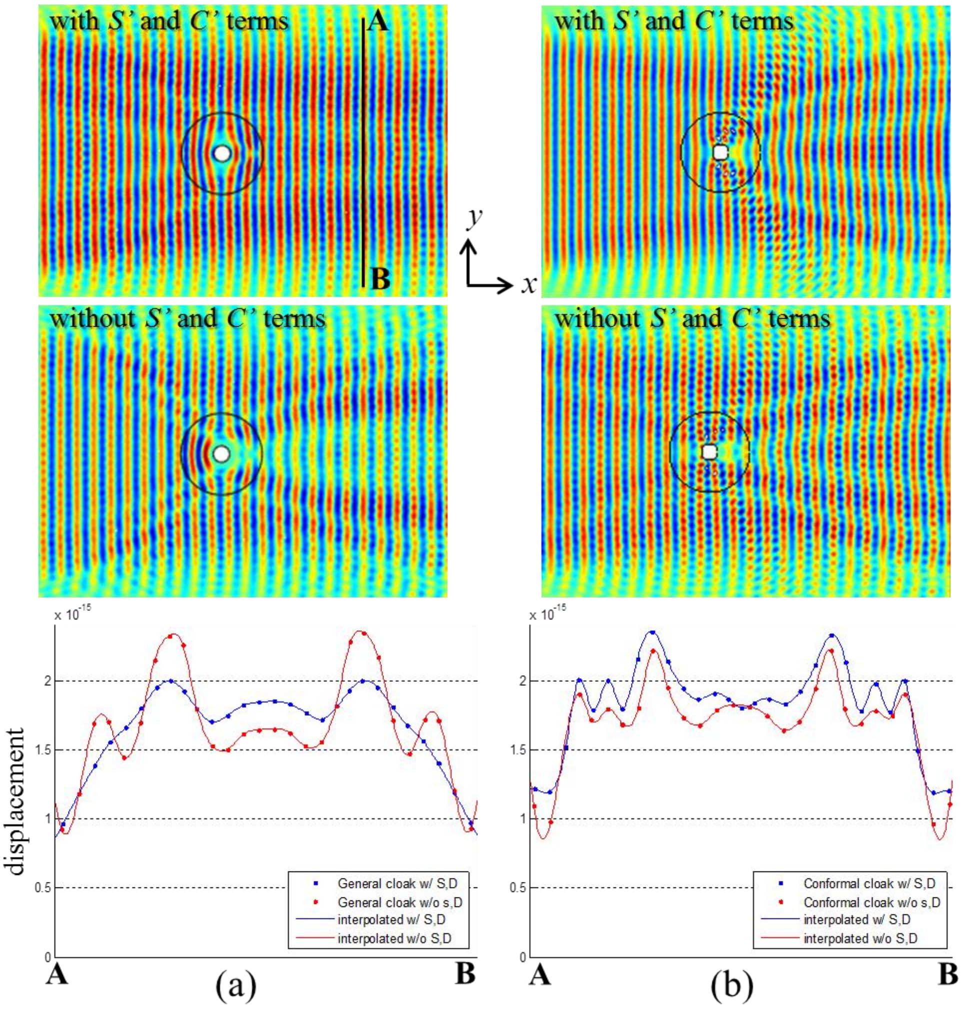

Figure S2. Displacement distribution in a plate (thickness t_p = 3 mm) with a hole covered by annular cloaks (inner radius a = 2 cm, outer radius b = 10 cm) obtained with (a) non-conformal (Eq. 22) and (b) conformal transformations (Eq. S23). The incident plane wave propagation along the +x axis was the lowest symmetric time-harmonic Lamb wave centered at 150 kHz. The lower plots for each case show the horizontal displacement components calculated along $\overline{AB}$.

In order to estimate the effect of ignoring the $\underline{S}'$ and $\underline{C}'$ terms quantitatively,

the values of the R_{xx}, R_{xy}, and R_{yy} measures are plotted in Fig. S3 for the region of $a < r < b$. Apparently, the value ranges for R_{xx}, R_{xy}, and R_{yy} for the conformal mapping were much smaller than those for the non-conformal mapping. As seen in Fig. S3, the minimum and maximum values of R_{xx}, R_{xy}, and R_{yy} appear only in small, localized regions. The effect of the ignoring the $\underline{S}'$ and $\underline{C}'$ terms is evaluated while introducing a single measure $\overline{R}$ that is the average of $\overline{R}_{xx}$, $\overline{R}_{xy}$ and $\overline{R}_{yy}$, each of which denotes the root mean squared value of R_{xx}, R_{xy}, and R_{yy}, respectively, over the annular cloak region $a < r < b$. The calculation shows that $\overline{R}$ = 0.230 ($\overline{R}_{xx}$ = 0.187, $\overline{R}_{xy}$ = 0.284, $\overline{R}_{yy}$ = 0.218) for the non-conformal mapping (S23) and $\overline{R}$ = 0.162 ($\overline{R}_{xx}$ = 0.144, $\overline{R}_{xy}$ = 0.271, $\overline{R}_{yy}$ = 0.0698) for the conformal mapping (S22). These results indicate that the magnitude of the $\underline{S}'u'$ term with respect to $\underline{C}'\nabla'u'$ is only 16% for the conformal mapping while that for the non-conformal mapping is 23%. Therefore, ignoring the $\underline{S}'$ and $\underline{C}'$ terms for the conformal mapping has a less significant effect for the overall wave field, as shown in Fig. S2.

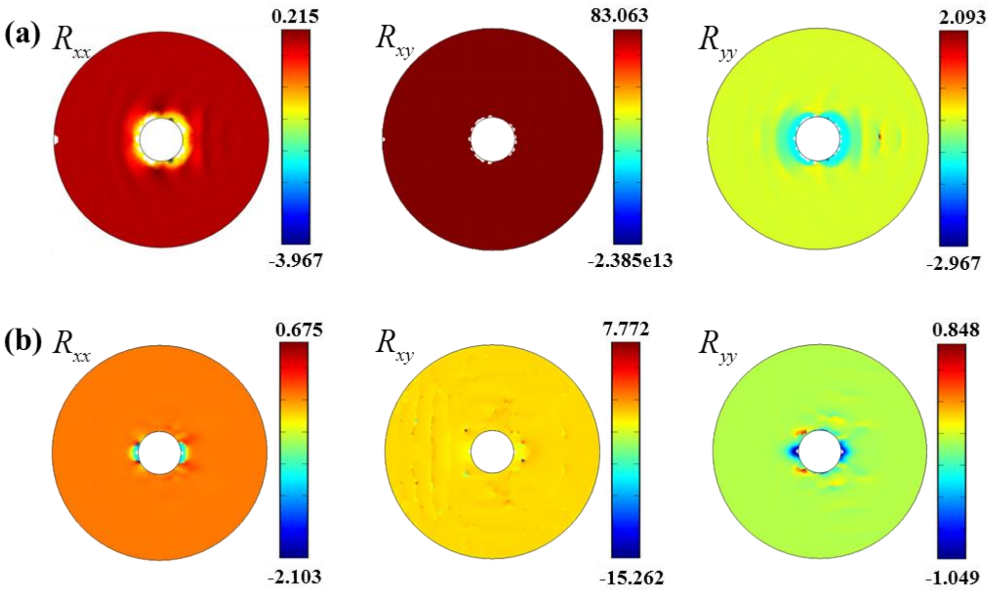

Figure S3. The distributions of R_{xx}, R_{xy}, and R_{yy} in the cloaking designed using (a) the non-conformal transformation (S22) having $\overline{R}$ = 0.230 ($\overline{R}_{xx}$ = 0.187, $\overline{R}_{xy}$ = 0.284, $\overline{R}_{yy}$ = 0.218) and (b) the conformal transformation (S23) having $\overline{R}$ = 0.162 ($\overline{R}_{xx}$ = 0.144, $\overline{R}_{xy}$ = 0.271, $\overline{R}_{yy}$ =0.0698). Thus, the contribution of the $\underline{S}'u'$ term with respect to $\underline{C}'\nabla'u'$by the conformation mapping is considerable smaller than that with the non-conformation mapping.

References

R1. M. Kadic, T. Buckmann, N. Stenger, M. Thiel, M. Wegener, On the practicability of pentamode mechanical metamaterials, Appl. Phys. Lett. 100 (2012) 191901.

R2. A. N. Norris, A. L. Shuvalov, Elastic cloaking theory, Wave Motion 48 (2011)

525-538.

R3. R. Schinzinger, P. A. A. Laura, Conformal mapping: methods and applications, Dover Publications Inc., Mineola, NY (2003).

R4. Y. Urzhumov, N. Landy, D. R. Smith, Isotropic-medium three-dimensional cloaks for acoustic and electromagnetic waves, J. Appl. Phys. 111 (2012) 053105.
